# The Applicability of Self-Play Algorithms to Trading and Forecasting Financial Markets

**DOI:** 10.3389/frai.2021.668465

**Published:** 2021-05-31

**Authors:** Jan-Alexander Posth, Piotr Kotlarz, Branka Hadji Misheva, Joerg Osterrieder, Peter Schwendner

**Affiliations:** ^1^School of Management and Law, Institut für Wealth & Asset Management, Zurich University of Applied Sciences, Winterthur, Switzerland; ^2^School of Engineering, Institute of Data Analysis and Process Design, Zurich University of Applied Sciences, Winterthur, Switzerland; ^3^Department for Finance, Institute for Finance, University of Liechtenstein, Vaduz, Liechtenstein; ^4^The Hightech Business and Entrepreneurship Group, Faculty of Behavioural, Management and Social Sciences, University of Twente, Enschede, Netherlands

**Keywords:** artificial intelligence, self-play, machine learning, financial markets, trading

## Abstract

The central research question to answer in this study is whether the AI methodology of Self-Play can be applied to financial markets. In typical use-cases of Self-Play, two AI agents play against each other in a particular game, e.g., chess or Go. By repeatedly playing the game, they learn its rules as well as possible winning strategies. When considering financial markets, however, we usually have one player—the trader—that does not face one individual adversary but competes against a vast universe of other market participants. Furthermore, the optimal behaviour in financial markets is not described *via* a winning strategy, but *via* the objective of maximising profits while managing risks appropriately. Lastly, data issues cause additional challenges, since, in finance, they are quite often incomplete, noisy and difficult to obtain. We will show that academic research using Self-Play has mostly not focused on finance, and if it has, it was usually restricted to stock markets, not considering the large FX, commodities and bond markets. Despite those challenges, we see enormous potential of applying self-play concepts and algorithms to financial markets and economic forecasts.

## Introduction

Over the years, academics and experts in computer science and statistics have developed advanced techniques to obtain insights from large data-sets combining different data types obtained from a variety of sources (see Brito, 2014). These models can utilise the ability of computers to perform complicated tasks by learning from experience. Following a definition offered by the Financial Stability Board (2017). Artificial Intelligence (AI) is a broad term capturing “the application of computational tools to address tasks traditionally requiring human sophistication.”

It is essential to mention that often, the terms AI and Machine Learning (ML) are used interchangeably. However, AI is a broader term, of which ML represents a subcategory: the difference being that ML is a data-driven way to achieve AI, but not the only one. Similarly, big data analytics is broader than ML, as it also includes statistical learning.

AI is getting more and more attention nowadays. Its practical application to various fields and possible benefits are changing business landscapes even in the most conservative areas. AI is currently applied in risk management, fraud detection, big data, and trading. In most cases, the literature offers empirical evidence of AI-based methods significantly outperforming other conventional approaches. Self-play algorithms belong to the area of AI, precisely ML, focusing on how agents ought to take actions in an environment to maximise some notion of cumulative reward. They are frequently associated with multi-agent Reinforcement Learning (RL). The term refers to that dynamic programming approach that aims to train algorithms using a system of reward and punishment. Nonetheless, a comprehensive definition is hardly available as the term is typically related to numerous algorithms and approaches. The main goal of the self-play concept is to achieve superhuman performance in many challenging tasks, such as games, decision-making processes and trading activities. Through numerous interactions with the environment, the algorithm (agent) learns without intervention from humans and maximises the reward function. The agent receives rewards when performing the correct decision and penalties otherwise. The idea behind reinforcement learning is initially inspired by behaviourist psychology. The algorithm learns similarly to a child performing a new task (Sutton and Barto, 2018).

## The Applicability of Self-Play Algorithms to Trading and Forecasting Financial Markets

When contemplating the conceptual transfer of self-play algorithms to their applications in financial markets, we are immediately confronted with two fundamental challenges:

(i) The information space based on which decisions are made is unbounded and, to some extent, unknown.
(ii) In contrast to classical self-play exercises, either one player is involved (one agent learning against the backdrop of a pre-determined market environment) or a myriad thereof (the multitude of agents constituting the market themselves).

Before addressing these challenges in detail, let us first look at why self-playing AI agents are still a viable candidate for financial decision-making tasks as well.

AI methods employing self-play algorithms have recently been very successful in mastering a number of challenges difficult to overcome by conventional machine learning approaches, i.e., challenges characterised with high complexity of the problem-set (high dimension of phase space, non-linear causality), incomplete knowledge of the (defined and bounded) information set, necessity for real-time, ambiguousness of the solution space, etc.

Self-play algorithms have a long history in playing traditional games, such as chess, checkers, backgammon, and Go (Samuel, 1959; Tesauro, 1992; Silver et al., 2017, 2018). Samuel (1959) created an advanced program to play checkers using so-called “alpha-beta” pruning and several forms of forward-pruning to restrict the spread of the move tree allowing the program to look deeper ahead. Even though the program was unable to outplay checker masters, its playing ability has been relatively high, compared with other existing approaches. Another example comes from Tesauro (1992) who designed a neural network to play backgammon based entirely on the self-play board configuration. It is also worth noting that the backgammon game also comprises an element of randomness induced by the dice role in the play.

Silver et al. (2017) and Silver et al. (2018) provide a comprehensive report on well-trained AI agents like DeepMind's AlphaGo or AlphaStar which have mastered various critical aspects of such games and succeeded in competing against world-class human players, consistently beating them and even inventing new strategies previously never employed by human players. The recently developed AlphaStar AI, the AlphaZero taught to play Starcraft II, Dota and Poker consequently outperformed top game players (Silver et al., 2017; Wang et al., 2018).

However, it is essential to note that for successfully playing Starcraft II, in addition to fast information processing and computation of complex decision trees, qualities like mid- to long-term strategic planning, creativity, dealing with ambiguousness, and capability to adapt one's behaviour to a changing environment, are necessary—qualities so far only attributed to human players. Nevertheless, it seems possible to teach and to breed AI agents, that can accomplish quite remarkable feats—at least in the predefined and bounded environment of a game.

Let us now turn our attention to financial markets. Badea (2000) is one of the very first, successful attempts to apply the Inductive Logic Programming (ILP) for combinations of well-known technical indicators based on historical trading data. The author identifies the ideal trading opportunities and feeds them to the ILP learner, which consequently produces trading strategies with clearly identifiable rules, as an output. Halperin and Feldshteyn (2018) propose a completely new method for signals, based on the self-learning approach, which could be considered an extension of the well-known Black-Litterman model, that remains one of the most important approaches in portfolio management because of its simplicity and strict focus on market dynamics. The Bounded Rational Information Theoretic Inverse Reinforcement Learning (BRIT-IRL) model developed by Halperin and Feldshteyn (2018) captures market dynamics and unknown patterns from stock market data.

Nowadays, there exist a few promising attempts to apply the self-play algorithms to trading. In 2018, Edward Lu developed a deep reinforcement learning model Q-Trader.^1^ The model was supposed to achieve stock trading short-term profits and has been tested on the S&P 500 index giving statistically significant positive results. However, in terms of long-term decision making, it was not as suitable as when applied to shorter periods. The Q-Trader uses an exciting concept called experience replay, which is very similar to the AlphaGo strategy developed by DeepMind.

Furthermore, the academic literature offers another exciting attempt to apply the AlphaGo strategy to financial markets,^2^ in particular to stock trading and to asset pricing, i.e., how companies' financial performance impacts equity prices. Although this research is still in early stages and there are numerous open questions, considering its past successful performance (AlphaGo possesses the highest possible Go ranking), it is without a doubt that trading strategies based on techniques similar to those used in AlphaGo, have the potential to significantly impact financial markets and optimal trading strategies. The proposed trading system would require a deep neural network specification.

It is crucial to note that the majority of current approaches focus exclusively on stock markets. Other markets such as FX, commodities and bond markets seem to be significantly unresearched, offering ample space for further research and analysis. The stock market prediction indeed has solid fundamentals, meaning numerous prediction models are giving interesting starting points.

Furthermore, FX and commodity markets are frequently considered as drivers of the global economy and international trade. The prices of strategic commodities such as oil, metals, and gas have a massive impact on economies in terms of inflation, government spending or foreign direct investments. The strength or weakness of major currencies significantly affects international trade. Hence, investigating what drives the global economy seems to be imminent in such an analysis. However, the successful application of self-play algorithms to all asset classes will be a remarkable achievement that might completely transform the current state of trading.

Furthermore, multi-agent RL can help to model decisions made under the theoretic framework of game theory and hence make the process understandable, transparent, and explainable. Modelling the behaviour of AI agents as they decide how to behave under certain risk/reward target functions is therefore not limited only to trading activity but easily can be also applied to e.g., credit approvals, sales/customer interaction, risk management, financial negotiations, and drivers for systemic risk (like credit spreads, volatility, sovereign bond spready, EM currencies).

## Major Challenges in the Application of Self-Play Algorithms

We group the major challenges into three main categories: (i) data challenges (ii) challenge of players and (iii) modelling and simulation issues. We provide an overview of existing solutions to those three topics.

### Data Challenges

The academic research has somehow stayed away from financial markets due to numerous reasons. On the one hand, the availability and selection of data constitute a substantial challenge. On the other hand, financial markets are inherently chaotic and frequently considered as unpredictable, hence efficient (Mussa, 1979; Meese and Rogoff, 1983; Lipton-Lifschitz, 1999). Indeed, financial markets are typically determined by a substantial number of time-dependent processes and factors, which are also non-stationary. Hence, building an adequate prediction model capable of simultaneously capturing all factors, processes and the evolution of markets is often not possible. This task becomes further burdened by the rapid changes that characterise financial markets.

It is worth noting that the high complexity of financial markets and a large number of potential industry drivers might lead to model selection and over-fitting issues. On the other hand, the availability of historical data also remains a significant challenge. The financial markets change even on micro- to milliseconds time scales, and many macroeconomic factors are available only on weekly, monthly or in some cases on a quarterly frequency. For instance, nowadays, in many cases, it seems that stock prices are more influenced by the unavoidable daily noise included in media coverage than by the companies' actual performance and thus the separation of the signal from the noise is one of the major challenges when dealing with financial data.

Furthermore, the risk of not having sufficiently large data sets for training AI models remains relatively high. Therefore, to train models, it might be necessary to generate more data by doing simulations. Three established practises of achieving this objective are: (i) employing stochastic processes (Janke, 2007), (ii) constituting the market through players that by themselves create more data and (iii) applying generative models such as GANs (Alqahtani et al., 2019).

Even in the above-mentioned restricted setting of, say, a momentum trader, this remains an arduous task. In the first instance (a stochastic process), we need to make sure that the statistical properties of the simulated data are aligned with the historical data and consistent across the market at each point in time. In the second (players simulate their own market) and third (generative models) instance, the same holds true. The statistical properties of the simulated data need to match the historical market, need to be consistent in themselves, and need to follow economic rationale—in short, whatever is simulated needs to “look and feel” like a real market.

### Challenges Related to the Number of Participants

Here we discuss and analyse the challenges related to the particular situation in financial markets, where we either have only one player or a myriad thereof.

When thinking about this second challenge, it might make sense to employ an analogy to Statistical Thermodynamics and its origins. Starting from a free-moving one single particle, we can efficiently compute its trajectory. Furthermore, we know that as long as no force is acting on it, it will not change its state of motion. This is a rather dull situation, akin to one single trader: he cannot trade, no matter how many tradeable assets he has at his disposal, simply because there is no one to trade with.

Let us add another particle: Now, we can still compute the trajectories of both particles and even their interactions. Analogous, our two traders can interact and trade. However, the results will be quite boring in the case of particles and probably either non-existent or very strange in the case of two traders. Only when we add more and more particles, things become interesting: Now, it is no longer of any use describing the trajectories of all the single particles, but a different behaviour emerges that we can capture at a higher level *via* the associated statistics of the integrated aggregate.

We could assume, or indeed would hope, that something similar happens when simulating quite a large number of individual traders: By the interaction of all the traders in the virtual market, a top-level market behaviour emerges that is—if we have done things “right”—closely resembling the real market. Furthermore, if we proceed to the limit of an infinite number of traders, from the viewpoint of one single (small) trader, all the other traders will most probably “look like a continuum,” i.e., like “the market.”

Consequently, one single trader/player should not be able to discern whether the market he is facing is simulated based on a top-down approach (e.g., employing stochastic processes) or based on a bottom-up approach (e.g., by the interaction of a myriad of single traders). As long as the single player does not in any noticeable way influence and move the market, this leaves us with the following interesting conclusions:

(i) When training a single AI agent against the market, it should make no conceptual difference whether this market is based on real historical data or simulated data, be it *via* stochastic processes or a multi-trader ecosystem: The AI agent will experience an infinitely deep market that dictates its trading environment.
(ii) In the case of the simulated multi-trader ecosystem, we then would train in self-play not one or two but a substantial number of AI agents in parallel. This approach could prove very efficient concerning strategy formation and trading optimisation.
(iii) As soon as the trading actions of single AI agents become dominant enough to provoke measurable feedback for the market (e.g., a single AI agent starts to actively “move the market”), we should be able to observe a phase transition: the entirety of traders suddenly decomposes into “market-movers” and “non-market-movers.”
(iv) In the case of the multi-agent system with a small number of archetypes of macro players (AMP)—such as the largest central banks (Fed, ECB, BOJ, BOC), the largest Federal/supranational governments (US, EC, Japan, China) and the largest private sector players (US banks, US corporates, US households, EU banks, EU corporates)—the marginal behaviour of each of these players as a function of market and macro data could be modelled. The model for AMPs would then calibrate to multivariate data and collective past behaviour. By this, the AMPs would function as external market boundary conditions with regard to the other (smaller) AI agents. A model structured like this could be useful to forecast any political or market reactions to unilateral actions, e.g., policy changes to tariffs.
(v) The AMP concept could also be useful to forecast correlated “risk-off” market movement patterns (Papenbrock and Schwendner, 2015) characterised by a liquidation of carry trades in all asset classes, leading to a sharp drawdown in risky assets, a capital flight into the highest rated sovereign bonds (Broner et al., 2010) and a devaluation of emerging market currencies against the funding currencies USD, JPY, CHF, and EUR, an increase in credit spreads and volatility spikes especially in equities. The “non-linear” reaction of markets is amplified by pro-cyclical risk management systems comparing the current realised volatility with long-term historical volatility and forcing to unwind positions in stressed situations (Packham et al., 2017). These risk management systems are prevalent both in banks in the form of (conditional) value-at-risk and at investment funds in the form of target volatility concepts (Jaeger et al., 2020).

From the conclusions made above, research questions immediately present themselves:

— How many agents need to be simulated to constitute something that “acts and feels” like a real market?
— Should all of these AI agents start alike, or should initial conditions be different (different classes or styles of AI agents)?
— How should one deal with “market movers:” Should they be restricted in some way, completely unrestricted, or perhaps even exogenously given or deterministically modelled?
— How and to which extent should we provide the AI agents with exogenous data, e.g., macro variables? Or conversely, can a market be simulated at all when only price data is available to the agents?
— Moreover, how would this restrict the learning process of the self-players?

The simulation of a market based on the actions of many single AI agents also has exciting implications with regard to understanding market dynamics and could potentially deliver insight far beyond robust forecasting and optimal trading strategies. On the other hand, hybrid approaches may offer a way to reduce complexity. As an example, we can think about providing some market parameters (e.g., macro data, news, and suchlike) “externally” as given while the AI agents still simulate the price dynamics by trading.

Finally, a selection of rule-based trading styles as agents that generate not only signals but also forecasts of global asset flows stemming from these trading styles might yield additional insights into overall market behaviour.

### Challenges Related to Modelling and Simulation

For the simulation of a multi-agent model, there are several platforms such as Netlogo, Agent Sheet, Ascape, Repast, Mason, Anylogic, Flame, Swarm, Starlogo (Souissi et al., 2018), which can be used in the context of this work as well.

No matter which approach we choose, we need to infer rules and algorithms sensibly describing the market. This is already very complex if we only look at price data and becomes probably unmanageable once we decide to include additional information, like macro data or news flow.

As a side note, we remark that even when just referring to historical data, providing information beyond prices becomes a challenge. For each point in time, we would have to constitute the full set of data available just then—a virtually impossible task: consider the case of including news flows.

The literature offers several works that have employed multi-agent modelling and simulation, including Ehrentreich (2003), Kumar et al. (2010), and Naciri and Tkiouat (2016). The most recent attempt to a multi-agent simulation of the stock market is proposed by Souissi et al. (2018). Namely, the authors simulate a simplified stock exchange with three types of investors (zero intelligent trader, fundamentalist trader and traders using historical information in the decision-making process) and one type of asset, to analyse the evolution of traded volume on exchanges depending on the type of investor. Similarly, as in most available research, the three agents in Souissi et al. (2018) model interact with each other and make decisions based on a number of rules. The results indicate that financial markets' stability and performance is strongly impacted by the distribution of the types of traders and the introduction of imitation mechanisms. Finally, it is unclear what the full set of information is that the market players are looking at when deciding on trades. Furthermore, the issue becomes even more complex in view of different asset classes where a completely different set of information drives the forecasts and market actions, i.e., within each asset class, the market participants “function” significantly differently with regard to the data considered. This observation brings us back to our first challenge, i.e., the infinite and, in parts, unknown information space. Therefore, no matter which way we address the task of providing a playing field for the AI agents (as we will see later), by referring to historical data or by simulating the market environment, we are left with a stunning complexity and with many choices to make when defining the playing field at each instant. A possible solution to this might be to limit the necessary information content by restricting ourselves to certain, well-defined trading/investment styles, e.g., momentum traders (“price data only”). Of course, this raises the important question what exactly the relevant information content for a specific trading style is and immediately presents one with the next challenge: Being unaware of relevant information and/or deliberately excluding it for the sake of simplicity will compromise or at least bias the quality of the results obtained.

## Discussion and Conclusion

AI's ability to significantly outperform other well-known conventional methods makes it one of the fastest- growing areas in our rapidly changing world. Self-play algorithms, as an area of AI, possess a broad definition offering substantial space for the application of various machine learning approaches. In its essence, self-play algorithms focus on how agents should act in an environment, so to maximise some defined cumulative reward function.

Despite the great importance of AI for risk management, big data analysis, credit risk and fraud detection, the application of self-play algorithms to financial markets seems to be underexploited in terms of both academic and industry-related research. The usage of self-play algorithms in trading is highly challenging, requires large data sets, multiple simulations and scenarios.

Within this report, we provide a detailed description of existing methods to deal with data challenges arising when using AI techniques in financial markets. Besides, we analyse the challenges related to the number of market participants involved in financial markets and the potential ways of modelling that. Furthermore, we identify the main academic articles applying self-play algorithms in financial decision- making tasks, which can be used as a starting point for broader research.

In terms of the practical application of self-play algorithms, there exist two major applications performed by the well-known companies Bloomberg and DeepMind. The latter one (the developer of AlphaZero and AlphaGo) attempts to create a securities trading system for stocks similar to the AlphaGo algorithm.

Furthermore, there is substantial potential for further research when entering asset classes other than equities, such as FX, commodities and bond markets, which seem to be significantly under-researched, or when contemplating financial forecasting challenges beyond trading, such as e.g., credit approval processes or drivers for systemic risk (like credit spreads, volatility, sovereign bond spready, EM currencies).

To summarise, the practical application of self-play algorithms to financial markets and trading is undoubtedly a challenging task. However, the prospective benefits from truly well-performing trading strategies and the substantial contribution to the academic research regarding forecasting and facilitating a deeper understanding are making this topic highly relevant.

## Author Contributions

All authors contributed equally to the research, writing of the manuscript, read, and agreed to the published version of the manuscript.

## Conflict of Interest

The authors declare that the research was conducted in the absence of any commercial or financial relationships that could be construed as a potential conflict of interest.

## Footnotes

^1^Deep Q-learning driven stock trader bot, Github: https://github.com/edwardhdlu/q-trader.

^2^“AlphaGo for Securities Trading,” Tod Moses, June 2018, https://medium.com/todd-moses/alphago-for-securities-trading-d37854847e48.
